# Obstructive nephropathy requiring dialysis secondary to genitourinary tuberculosis with concomitant pulmonary tuberculosis in an immunocompetent young adult: A case report

**DOI:** 10.1016/j.idcr.2026.e02801

**Published:** 2026-10-14

**Authors:** Henok Taddese Ghebremichael, Amanuel Dagabas Wakoya, Mikiyas Wondwesen Ayele, Dagmawi Antneh Teferi, Tadege Haregewoine Alemayehu

**Affiliations:** a Department of Internal Medicine, St. Paul's Hospital Hospital Millennium Medical College, Addis Ababa, Ethiopia; b Department of Intensive care Medicine, St. Paul's Hospital Millennium Medical College, Addis Ababa, Ethiopia; c Department of Emergency and Critical Care Medicine Haramaya University, Harar, Ethiopia; d Department of surgery, Urology Division, St. Paul's Hospital Millennium Medical College, Addis Ababa, Ethiopia

**Keywords:** Urogenital tuberculosis, Obstructive uropathy, Pulmonary tuberculosis, GeneXpert MTB/RIF, Immunocompetent adult,Case report

## Abstract

**Background:**

Genitourinary tuberculosis (GUTB) is a common form of extra-pulmonary tuberculosis that often presents with nonspecific urinary symptoms and is frequently diagnosed late. Genitourinary tuberculosis can cause progressive urinary tract obstruction, hydronephrosis, and renal impairment, particularly when diagnosis is delayed. Concurrent pulmonary involvement may occur and should be considered when evaluating patients with suspected genitourinary tuberculosis.

**Case presentation:**

A 39-year-old male presented with a two-year history of progressive lower urinary tract symptoms,later accompanied by hematuria, constitutional symptoms, and altered mentation. Laboratory evaluation revealed severe anemia, elevated inflammatory markers, and progressive acute kidney injury. Abdominal CT demonstrated chronic right renal atrophy with left hydroureteronephrosis with ureteric wall thickening, asymmetric bladder wall thickening, and significant abdominopelvic lymphadenopathy. Urine GeneXpert MTB/RIF was positive for Mycobacterium tuberculosis. Chest radiography and HRCT confirmed concurrent active pulmonary tuberculosis with apical cavitary lesions and tree-in-bud nodules. The patient required hemodialysis for refractory hyperkalemia and uremic encephalopathy. After stabilization, renal-dose antituberculous therapy (RHZE) was initiated the patient showed progressive clinical and renal improvement. He was discharged and continued antituberculous therapy.

**Conclusion:**

This case highlights that obstructive uropathy and renal failure may be presenting manifestations of genitourinary tuberculosis even in immunocompetent adults living in high-burden settings. Concurrent pulmonary tuberculosis should therefore be actively investigated. Early use of urine-based molecular testing, such as GeneXpert, together with a high index of suspicion may facilitate timely diagnosis and treatment, potentially preventing irreversible renal and urinary tract damage.

## Highlights

• Obstructive nephropathy requiring dialysis can be an initial presentation of genitourinary TB.
• Concurrent pulmonary and genitourinary TB can occur in immunocompetent adults.
• Urine Xpert MTB/RIF can facilitate rapid diagnosis of genitourinary TB.
• Antituberculous therapy, dialysis, and supportive care were associated with good recovery in genitourinary TB.

## Introduction

Tuberculosis (TB) remains a major global health challenge. According to the World Health Organization, more than 10 million people develop TB each year and over one million die from the disease, making it the leading cause of death from a single infectious agent [1]. Although pulmonary tuberculosis is the most common form, extrapulmonary tuberculosis (EPTB) accounts for a substantial proportion of cases and can involve virtually any organ system.

Genitourinary tuberculosis is one of the most frequent forms of EPTB. It typically results from hematogenous dissemination of Mycobacterium tuberculosis from a primary pulmonary focus to the kidneys, prostate, or epididymis. From the kidneys, infection may spread descending through the collecting system to the ureters, bladder, and urethra, and via the ejaculatory ducts to the genital organs [2], [3]. GUTB occurs across all age groups but is most common in men in their fourth and fifth decades of life. The disease is often insidious, with a long latent period (ranging from 5 to 40 years) between the initial pulmonary infection and the appearance of clinically evident urogenital disease. As a result, symptoms usually appear only at an advanced stage, leading to diagnostic delay and irreversible organ damage, including renal failure in some patients [2].

Clinically, GUTB lacks specific features. Most patients present with nonspecific lower urinary tract symptoms such as frequency, dysuria, urgency, sterile pyuria, hematuria, or flank pain. These manifestations frequently mimic bacterial urinary tract infection, resulting in repeated courses of antibiotics without lasting improvement. Constitutional symptoms (fever, night sweats, weight loss) are often absent unless concurrent pulmonary or disseminated disease is present. Because of its subtle and atypical presentation, a high index of suspicion is required, particularly in individuals living in or originating from high-TB-burden regions [2], [3].

Early diagnosis is essential to prevent progressive destruction of the urinary tract. Advances in molecular diagnostics, especially the Xpert MTB/RIF assay performed on urine, have significantly improved the speed and accuracy of detection. Nevertheless, many cases continue to be diagnosed late, when obstructive uropathy, hydronephrosis, or renal impairment have already developed.

Here we report a case of severe bilateral obstructive uropathy secondary to genitourinary tuberculosis with simultaneous pulmonary involvement in an immunocompetent adult. This case highlights the diagnostic challenges of Genitourinary tuberculosis, the value of urine molecular testing, and the importance of considering tuberculosis even in immunocompetent patients presenting with chronic urinary symptoms and progressive renal dysfunction.

## Case presentation

A 39-year-old man presented with a two-year history of progressive difficulty in urination and dysuria. During this period, he had been evaluated at a local general hospital, where he was managed with urinary catheterization and unspecified medications. He subsequently presented to our hospital with worsening urinary difficulty over the preceding two weeks, accompanied by reddish discoloration of the urine, purulent urethral discharge, and dysuria. His renal function had progressively deteriorated, and he subsequently developed confusion and altered mental status. He also reported loss of appetite, significant unquantified weight loss, and night sweats.

On examination, the patient was acutely ill-looking. His blood pressure was 104/70 mmHg, pulse rate 102 beats/minute, respiratory rate 28 breaths/minute, and oxygen saturation 90% on room air, with a BMI of 21.6 kg/m². Chest examination revealed fine crepitations with decreased air entry over the lower one-third of both lung fields and coarse crepitations over the right upper lung zone. The abdomen was soft,but he had left side costovertebral angle tenderness. A urinary catheter was in situ, but there was no urine output. His Glasgow Coma Scale score was 14/15 (E4V4M6)

## Investigations

Initial laboratory investigations revealed a white blood cell count of 4.72 × 10 ³ /µL, neutrophils 77%, hemoglobin 4.4 g/dL, and platelet count 122,000/µL. Serum creatinine and urea were 5.98 mg/dL and 93.8 mg/dL, respectively, and subsequently increased. Serum electrolytes were sodium 137 mmol/L, potassium 4.32 mmol/L, and chloride 105.4 mmoL Phosphorius,1.62(o.95–2.5)RBS and Hgba1c: 98 mg /dl and 5.4 Mg/dl. And the chronological findings are listed in (Table 1) Screening for HIV, hepatitis B surface antigen, hepatitis C, and syphilis by VDRL was negative. The erythrocyte sedimentation rate was elevated at 123 mm/hour. Urine Gram stain was negative, and subsequent urine culture did not reveal a causative organism. Initially, acute kidney injury secondary to possible sepsis-associated acute tubular necrosis was considered. However, because of the patient's persistent renal dysfunction, flank tenderness, and obstructive urinary symptoms, further imaging was performed.

**Table: 1:** Trend of laboratory investigations during admission.

Date	WBC (4–11 ×10 ³/µL)	Hgb (12.7–17.1 g/dl)	Hct (34.4–48.3%)	Na⁺ (136–145 mmol/l)	K⁺ (3.5–5.1 mmol/l)	Cl⁻ (98–107 mmol/l)	Creatinine (0.7–1.2 mg/dl)	Urea (16.6–48.5 mg/dl)
Before admission	4.72	4.4	15.0	129.0	3.52	96.0	5.98	93.6
25 Apr 2026	6.44	6.3	–	136.8	5.27	106.7	13.2 *	155.0
02 May 2026	–	–	–	–	–	–	9.71 #	139.3
03 May 2026	–	6.2	17.8	130.0	5.38	104.7	8.6	150.9
04 May 2026	3.95	6.9	19.5	134.0	5.13	103.1	–	–
06 May 2026	–	6.0	17.4	131.0	5.05	99.0	4.31	185.2
07 May 2026	–	–	–	133.0	5.44	100.2	2.2	–
09 May 2026	–	7.0	20.0	131.0	5.2	104.7	1.5	46.0

Other investigations: PICT non-reactive; HbA1c 5.4%; HBsAg negative; sputum GeneXpert positive; urine GeneXpert positive.

* -Dialysis started, # -stent inserted;PICT: provider-initiated counselling and testing (HIV); HbA1c: glycated haemoglobin; HBsAg: hepatitis B surface antigen.

Abdominal computed tomography demonstrated chronic right renal atrophy with marked cortical thinning and hydronephrosis, consistent with long-standing obstructive or reflux-related nephropathy. There was also left-sided hydroureteronephrosis with irregular pelvic and ureteric wall thickening. Asymmetric urinary bladder wall thickening, measuring up to 1.3 cm, was also observed. Significant abdominopelvic lymphadenopathy involving the peripancreatic/portal, para-aortic, and aortocaval regions was present, raising concern for a granulomatous disease (Fig. 1).

**Fig. 1:**
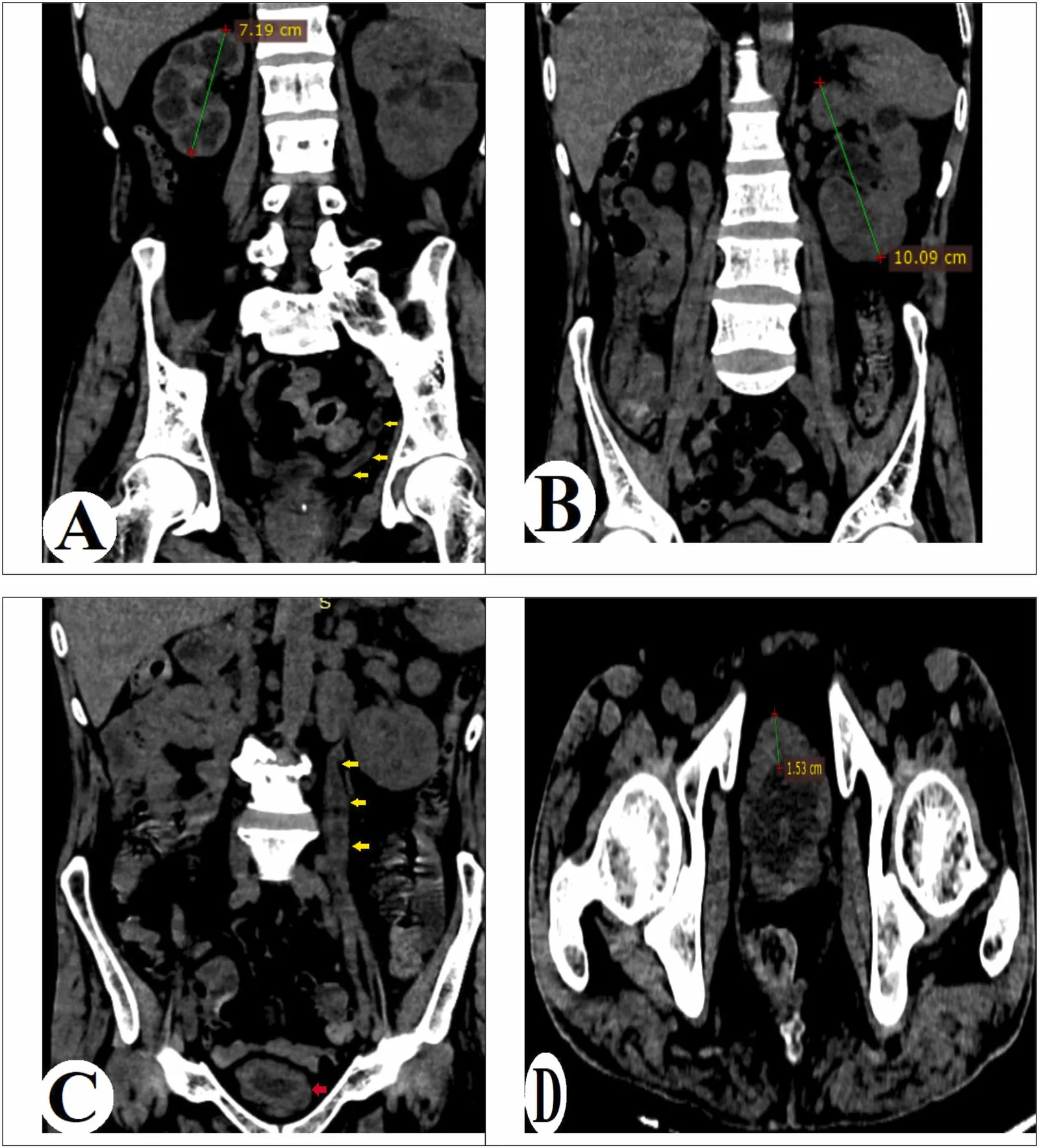
Abdominopelvic CT scan without contrast showing A) Small thinned out right kidney with left side distal ureteric thickening depicted in arrow B) Left normal sized kidney with moderate hydronephrosis and irregular outline C) Dilated and thickened left side proximal ureter as shown in the arrow D) contracted urinary bladder with asymmetric wall thickening.

Given the radiological findings and clinical suspicion, Urine GeneXpert MTB/RIF testing was performed on one an early-morning first-void urine specimen and detected Mycobacterium tuberculosis without rifampicin resistance; mycobacterial culture of urine and tissue biopsy were not performed. Further evaluation for pulmonary involvement was subsequently performed. High-resolution computed tomography (HRCT) of the chest demonstrated multiple bilateral centrilobular pulmonary nodules with a tree-in-bud pattern, right upper-lobe cavitary lesion with surrounding consolidation, findings considered highly suggestive of pulmonary tuberculosis. Infection (Fig. 2).

**Fig. 2:**
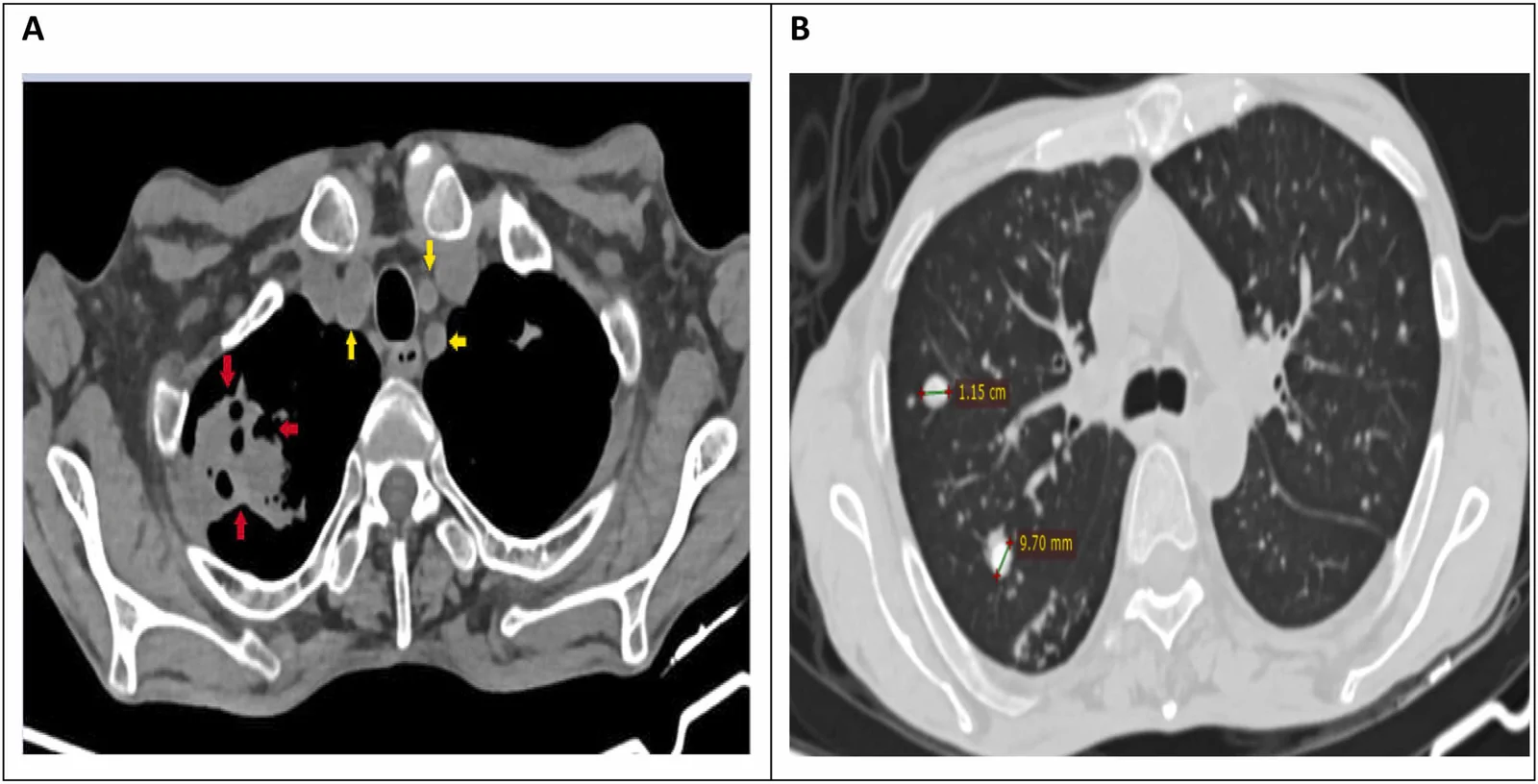
HRCT (A) Axial view Showing Multiple apical cavitary lesion (red arrows) with sorrounding ground glass opacity and multiple Lymphadenopathy(yellow arrow),(B) right mid lobe pulmunary nodules with largest measuring 1.1 cm.

## Treatment, outcome and follow-up

Following completion of the initial investigations, hemodialysis was initiated for refractory hyperkalemia and uremic encephalopathy secondary to acute kidney injury in the setting of obstructive uropathy. The patient received a total of seven hemodialysis sessions, with subsequent improvement in his mental status, renal function, and overall functional status. Given his severe anemia, he was also transfused with two units of compatible packed red blood cells. Following clinical stabilization, bilateral ureteric stenting was attempted to relieve the urinary obstruction; however, only left-sided ureteric stenting was successfully performed. After completion of hemodialysis and successful left-sided ureteric decompression, the patient remained clinically stable, with adequate urine output and continued improvement in renal function, with serum creatinine eventually stabilizing at 1.5 mg/dL. Antituberculous therapy was initiated with a renal-adjusted regimen: isoniazid 300 mg and rifampicin 600 mg were administered daily, while pyrazinamide 1800 mg (30 mg/kg) and ethambutol 1200 mg (20 mg/kg) were administered three times weekly immediately after hemodialysis sessions. Once renal function recovered and hemodialysis was no longer required, all four drugs were administered daily at standard doses. Pyrazinamide and ethambutol were discontinued after completion of the two-month intensive phase, and the patient continued isoniazid and rifampicin.At the time of follow-up, he was in his fourth month of antituberculous treatment and continued to attend outpatient follow-up. He demonstrated overall clinical improvement and remained on treatment, with a planned total treatment duration of six months. The chronology of the patient’s clinical course has been presented in (Table 2).

**Table 2:** Chronological sequence of clinical events and interventions.

Time point	Clinical status / key findings	Interventions / results
Hospital Day 1 (admission with pre admission Investigation from other hospital)	Anuria (catheter in situ), uremic encephalopathy (GCS 14/15), severe anemia (Hb 4.4 g/dL), creatinine 5.98 mg/dL (rising), hyperkalemia	Screened for HIV, hepatitis B surface antigen, hepatitis C, and syphilis, Urine Xpert was sent
Hospital Day 2	Cr= 13.2 urea = 155 with clinical uremic syndrome	Hemodialysis session 1
Hospital Day 3	Persistent anuria and uremic encephalopathy	Hemodialysis session 2
Hospital Day 4	Patient’s uremic symptoms persisted	Hemodialysis session 3
Hospital Day 5	The patient showed mental status improved	Hemodialysis session 4 (last of the consecutive daily sessions)
Hospital Day 7 (2 days after session 4)	Ongoing need for renal support Cr-9.71 urea-139.3	Hemodialysis session 5 and Bilateral ureteric stenting attempted; only the left ureter successfully stented (right side failed). Left-sided decompression achieved. 2 units PRBC transfused earlier during hospital course.
Hospital Day 9 (2 days after session 5)	Ongoing need for renal support	Hemodialysis session 6
Hospital Day 11 (2 days after session 6)	Ongoing need for renal support	Hemodialysis session 7 (final session)
Immediately after successful left stenting + clinical stabilization -on day 12	—	Renal-dose adjusted antituberculous therapy (RHZE, a initiated
Discharge	Clinically stable with residual renal function	Discharged on continued renal-adjusted ATT
Month 4 of ATT (outpatient follow-up)	Continued clinical improvement; no recurrence of obstruction or uremia	Still on treatment (planned total duration 6 months); creatinine remained stable

## Discussion

Genitourinary tuberculosis (GUTB) remains an under-recognized yet destructive form of extrapulmonary tuberculosis (EPTB), accounting for approximately 15–40% of EPTB cases and occurring in 2–20% of patients with pulmonary TB through hematogenous dissemination from a primary pulmonary focus [4], [5]. The kidneys are the most frequently involved site, with subsequent descending spread along the collecting system to the ureters, bladder, and urethra, and potential involvement of genital organs via the ejaculatory ducts. There is often a prolonged latent period (up to 20 years or more) between primary infection and clinically evident urogenital disease, during which dormant bacilli can reactivate, particularly in the setting of impaired cell-mediated immunity, although reactivation also occurs in immunocompetent hosts [4].

Our patient, a 39-year-old immunocompetent man, illustrates the classic but often overlooked natural history of GUTB. He presented with chronic lower urinary tract symptoms (frequency, urgency, dysuria, and episodes of acute urinary retention) over two years, initially managed as recurrent urinary tract infection or other common conditions. Constitutional symptoms (weight loss, night sweats, anorexia) appeared later and were accompanied by progressive obstructive uropathy culminating in severe bilateral hydronephrosis, renal atrophy, acute kidney injury requiring hemodialysis, and uremic encephalopathy. Imaging demonstrated the typical sequelae of advanced disease: chronic right renal atrophy with cortical thinning, left hydroureteronephrosis with irregular pelvic and ureteric wall thickening, asymmetric bladder wall thickening, and significant abdominopelvic lymphadenopathy—findings highly suggestive of granulomatous infection. Concurrent pulmonary involvement (apical cavitary lesions, tree-in-bud centrilobular nodules, and fibro-bronchiectatic changes) confirmed simultaneous genitourinary and pulmonary tuberculosis, a presentation that is well-documented yet frequently missed until late stages [4].

The pathogenesis explains these progressive changes. After hematogenous seeding of the renal cortex, bacilli preferentially multiply in the medulla (loop of Henle) because of local conditions of high ammonia concentration, hypertonicity, and relatively poor blood flow that impair host defenses. Granuloma formation, caseous necrosis, papillary destruction, fibrosis, and stricture formation ensue, leading to calyceal dilatation, hydronephrosis, and ultimately irreversible parenchymal loss or autonephrectomy (“putty kidney”). Ureteral strictures (most often distal) and bladder wall fibrosis further compound obstruction and reduce bladder capacity [2], [5]. In high-prevalence settings, such as many developing countries, clinicians must maintain a high index of suspicion for GUTB in any patient with sterile pyuria, persistent lower urinary tract symptoms unresponsive to conventional antibiotics, hematuria, or unexplained obstructive uropathy—even in the absence of immunosuppression [6].

Diagnosis is frequently delayed precisely because symptoms are nonspecific and conventional urine cultures are negative. Nucleic-acid amplification tests, particularly the Xpert MTB/RIF (or Ultra) assay performed on urine, have transformed early detection. Multiple studies demonstrate moderate-to-high sensitivity and near-perfect specificity of urine Xpert for GUTB, outperforming smear microscopy and offering results within hours rather than the weeks required for culture [7], [8]. In our case, a positive urine GeneXpert contributed to the diagnosis of genitourinary tuberculosis and supported the initiation of antituberculous therapy. Imaging (contrast-enhanced CT or CT urography, and HRCT of the chest) remains indispensable for defining the extent of organ damage, identifying concurrent pulmonary disease, and planning any necessary interventions [9].

Standard first-line antituberculous therapy—two months of isoniazid, rifampicin, pyrazinamide, and ethambutol followed by four months of isoniazid and rifampicin—is highly effective for drug-susceptible GUTB and achieves urine sterilization in the majority of patients within weeks [1]. Dose adjustment is required in renal impairment, as performed in this patient. While medical therapy is the cornerstone, advanced obstructive complications may necessitate temporary urinary diversion, stenting, or later reconstructive procedures or nephrectomy if a non-functioning kidney becomes a source of ongoing morbidity [6]. In our patient, hemodialysis supported recovery of renal function and mentation, transfusion corrected severe anemia, and attempted stenting was unsuccessful; nonetheless, clinical and microbiological improvement occurred with appropriately adjusted antituberculous treatment, allowing discharge and continued outpatient therapy.

This case underscores several important clinical lessons. First, GUTB can present with severe obstructive uropathy and renal failure even in young, immunocompetent adults living in endemic regions. Second, Concurrent pulmonary involvement may occur in patients with genitourinary tuberculosis and should be actively evaluated. Third, a high index of suspicion combined with modern molecular diagnostics (urine Xpert) and appropriate imaging can markedly shorten the diagnostic delay that historically leads to irreversible organ destruction. Finally, early initiation of standard therapy, renal support, and multidisciplinary care (nephrology, urology, infectious diseases, and pulmonology) offer the best chance of preserving residual renal function and achieving cure [2], [5].

In summary, obstructive uropathy of infectious etiology in high-TB-burden settings should prompt systematic evaluation for genitourinary tuberculosis. Recognition of this entity, even when it unmasks concurrent pulmonary disease, remains essential for timely intervention and prevention of lifelong morbidity.

## CRediT authorship contribution statement

**Henok Taddese Ghebremichael:** Writing – review & editing, Writing – original draft, Validation, Supervision, Formal analysis, Data curation, Conceptualization. **Amanuel Dagabas Wakoya:** Validation, Supervision, Conceptualization. **Mikiyas Wondwesen Ayele:** Validation, Data curation. **Dagmawi Antneh Teferi:** Writing – original draft, Conceptualization. **Tadege Haregewoine Alemayehu:** Supervision, Data curation.

## Consent for publication

Written informed consent for the publication of his clinical history and radio logical images has been obtained from the patient himself.

## Funding

None.

## Declaration of Competing Interest

The authors declare that they have no known competing financial interests or personal relationships that could have appeared to influence the work reported in this paper.

## Data Availability

Availability of data and materials Additional data that support the findings of this study are available on request from the corresponding author.
